# Inverse Association Between Metabolic Syndrome and Altitude: A Cross-Sectional Study in an Adult Population of Ecuador

**DOI:** 10.3389/fendo.2018.00658

**Published:** 2018-11-12

**Authors:** Amaya Lopez-Pascual, Jéssica Arévalo, J. Alfredo Martínez, Pedro González-Muniesa

**Affiliations:** ^1^Department of Nutrition, Food Science and Physiology, School of Pharmacy and Nutrition, University of Navarra, Pamplona, Spain; ^2^Centre for Nutrition Research, School of Pharmacy and Nutrition, University of Navarra, Pamplona, Spain; ^3^Nutrition Group, IdiSNA Navarra's Health Research Institute, Pamplona, Spain; ^4^CIBERobn Physiopathology of Obesity and Nutrition, Centre of Biomedical Research Network, ISCIII, Madrid, Spain; ^5^Madrid Institute of Advanced Studies (IMDEA Food), Food Institute, Madrid, Spain

**Keywords:** obesity, insulin resistance, hyperlipidemia, cardiovascular disease, type 2 diabetes mellitus, metabolic syndrome, high altitude

## Abstract

**Background:** Metabolic syndrome (MetS) is characterized by the clustering of hyperglycemia, hypertension, hypertriglyceridemia, low high-density lipoprotein cholesterol levels and central adiposity. Altitude has been proposed as a protective factor to prevent the development of MetS and its components.

**Aim:** To determine whether living at geographical elevation is associated with MetS and its individual components after adjustment for potential confounders in an Ecuadoran population.

**Methods:** The study included 260 Ecuadoran university graduates over 20 years of age, from the coastal or the Andean Altiplano region. The altitude of residence was imputed with the postal code of each participant residence according to the data of the Ecuadoran Geophysical Institute of the National Polytechnic School. MetS was defined according to the harmonizing definition. Logistic regression models were fitted to assess the relationship between altitude level and the prevalence of MetS and its individual components. To test the internal validity, re-sampling techniques were used (1,000 bootstrap samples).

**Results:** Living at high altitude was associated with less hypercholesterolemia (OR = 0.24; *p* < 0.001), hyperglycemia (OR = 0.25; *p* < 0.05) and MetS (OR = 0.24; *p* < 0.05), after adjusting for potential confounders. At high altitude the bootstrapped logistic regression models showed lower prevalence of hypercholesterolemia (OR = 0.30; *p* < 0.05), hyperglycemia (OR = 0.22; *p* < 0.001) and MetS (OR = 0.28; *p* < 0.05). The MetS score (0-5 points) showed a reduction in the number of MetS components at high altitude compared to sea level (B = −0.34; *p* = 0.002). A statistically significant lower self-reported energy intake was found in high altitude compared to sea level after adjustment for potential confounders (*p* < 0.001).

**Conclusion:** In the present study concerning a small Ecuadoran population composed of highly educated adults living at the coast and the Andean Altiplano, living at high altitude (2,758–2,787 m) was associated with a lower prevalence of MetS, hypercholesterolemia and hyperglycemia, compared to the participants at sea level (4–6 m). In addition, an inverse association between altitude and self-reported energy intake was found after adjusting for covariates, suggesting a physiological role of appetite at high altitude even in acclimated subjects.

## Introduction

The metabolic syndrome (MetS) is characterized by the clustering of hyperglycemia, hypertension, hypertriglyceridemia, low high-density lipoprotein cholesterol levels (c-HDL), and central adiposity (measured by waist circumference and body mass index) (1). The MetS is highly prevalent worldwide amounting to over 20% in Western countries (2). There is sufficient evidence indicating that MetS is a risk factor for cardiovascular disease and type 2 diabetes (3). Experts in clinical practice have published guidelines for the precise management of each risk factor in order to prevent or delay onset of type 2 diabetes and cardiovascular disease (1). Many risk and protective factors have been described to be associated to MetS, but there are contradictory outcomes among them (2). Therefore, research concerning the factors that are associated with the development of MetS is fundamental for its prevention and treatment.

Altitude has been proposed to increase cardiovascular and respiratory efficiency despite lower oxygen availability (4). Several observational studies have described that permanent highlanders had lower LDL cholesterol, higher HDL cholesterol and better fasting glucose levels as well as reduced obesity rates, when compared to sea level residents (4, 5). There is also evidence supporting lower blood pressure in subjects who reside at altitude, although results are inconclusive (4). In addition, epidemiological studies have reported lower obesity prevalence (6, 7) and incidence (8, 9), as well as lower diabetes (10) and hypertension rates (11). Moreover, a recent study published by our group showed lower incidence of MetS independent of risk factors and potential confounders (12). However, the results were recorded in a Spanish cohort, living at a moderate altitude [most of the people living below 1,500 meters (m)]. Other available epidemiological data have revealed that living at a higher altitude is associated with lower mortality from ischemic heart disease and certain types of cancer, and contrastingly a higher mortality rate from respiratory disease (13–15).

High altitude is commonly defined as living over 1,500 m (International Society for Mountain Medicine), while above 2,500 m people display a fall in arterial oxygen saturation (16). Living at high altitude represents a physiological challenge, thus, permanent residents present physiological adaptations to environmental pressure and lower oxygen availability such as raised hemoglobin concentration, enlarged lung volume and blunted hypoxic ventilatory response (5, 16). In this sense, three main high-altitude regions of the world characterize the successful human long-term adaptation to hypobaric hypoxia: the Ethiopian Siemen Mountains, the Tibetan Plateau in the vicinity of Himalayan valleys, and the Andean Altiplano (17). However, there is no common pattern of response to hypobaric hypoxia. Thus, the effects of altitude on the physiology of each population should be further investigated. In this context, the aim of this study was to determine whether geographical elevation is inversely associated with MetS and its individual components after adjustment for potential confounders in an Ecuadoran population. To our knowledge, this is the first study comparing an Andean population to those at sea level, with the objective to assess the association between altitude and MetS.

## Materials and methods

### Participants

The current study included a total of 260 Ecuadoran participants from Guayaquil and Triunfo at the coast region (sea level) and Riobamba at the Andean Altiplano (high altitude), where men and women (over 20 years of age) with a university degree were recruited from professionals of the National Police (District Triunfo-Bucay), the Ecuadoran Christian Center of Integral Theotherapy (Guayaquil and Riobamba) and teachers from the Middle School Chiriboga (Riobamba).

Participants were excluded if they were pregnant, had a body mass index (BMI) over 40 kg/m^2^ (morbid obesity), or suffered from cancer or cardiovascular disease. The study was conducted according to the guidelines laid down in the Declaration of Helsinki and it was approved by the Human Research Ethical Committee of the University of Navarra (code: 2018.011).

### Lifestyles and habits

Questionnaire data were collected by a dietitian-nutritionist, including information on each participant's characteristics and lifestyles (sex, age, smoking habit, alcohol consumption, exercise practice), as well as their anthropometric measurements (weight, height, waist circumference). A Harpenden anthropometric commercial tape was used to measure waist circumference (Holtain Limited, Crosswell, UK). Total energy consumption was calculated by 24-h dietary recall, of a representative day of the week, using dietary analysis software (Easy-Diet, Bicentury). The questionnaire included the postcode of each participant and the duration of their residence. Altitude of residence was imputed with the postal code according to the Ecuadoran Geophysical Institute of the National Polytechnic School.

### Assessment of metabolic syndrome and its components

Participants' BMI was recorded using self-reported weight in kilograms divided by the square of height in meters. Central adiposity was defined by a high waist circumference according to the Central and South American cut-off points 90 cm for men and 80 cm for women (3) or having overweight or obesity (BMI ≥25 kg/m^2^). MetS was defined according to the International Diabetes Federation and American Heart Association/National Heart, Lung and Blood Institute (IDF-AHA/NHLBI) harmonizing definition (1–3) which requires the diagnosis of three or more of the following five criteria (including central adiposity, aforementioned): Hypercholesterolemia (total cholesterol >240 mg/dl), hypertension (blood pressure: systolic ≥130and/or diastolic ≥85 mmHg), hyperglycemia (blood glucose ≥100 mg/dl) and low values of high-density lipoprotein cholesterol (HDL-c: men <40 mg/dl and women <50 mg/dl).

### Statistical analyses

Differences in characteristics of participants according to altitude level were evaluated with Pearson's χ^2^ test of linear trend for categorical variables or unpaired Student's *t*-test for continuous data. Logistic regression models were fitted to assess the relationship between altitude level and the prevalence of MetS and its individual components, categorized (yes/no) for the presence or absence of each risk factor. Odds ratio (OR) and 95% confidence intervals (95% CI) were estimated using as the reference category those participants who reside in the coast (Sea level). An initial model was analyzed including no covariates (crude). Following this, a model was fitted for sex and age only. The first multivariate model (Model 1) was fitted for potential confounding factors, including: age, sex, self-reported energy intake and duration of residence. A second multivariate model (Model 2) was additionally adjusted for: BMI, physical activity, smoking habit and alcohol consumption.

For internal validity, the risk of developing MetS living at high altitude was tested through re-sampling techniques (1,000 bootstrap samples) to derive adjusted OR and 95% CI. A multivariate linear regression model (Model 1) was used to assess the association between the altitude of residence and the summation of MetS components (MetS score: 0-5 points), where β-regression coefficients and their 95% CI were estimated using the sea level as the reference group. The differences between self-reported energy intake and altitude level were tested through analysis of covariance or ANCOVA (multiple adjustment with Model 1) to assess the influence of altitude on appetite after adjusting for potential confounding factors. The Stata 12.0 software (Stata, College Station, TX, USA) was used for all statistical analyses. Values of *p* < 0.05 were considered statistically significant.

## Results

### Subject characteristics

Two hundred and sixty participants were included in the study. Of these, 152 lived at sea level and 108 at high altitude. When comparing both altitude groups, those at high altitude were older, included a greater proportion of women, had lower self-reported energy intake and higher residence time (Table 1). BMI, physical activity, smoking habit and alcohol consumption were not significantly different between groups. The variables that were significantly different between groups were used in Model 1 as covariates, the rest of the variables were included in multivariate Model 2.

**Table 1:** Characteristics of subjects according to their altitude of residence.

**Elevation**	**Sea level**	**High altitude**	***p*-value**
Range (m)	4–6	2,758–2,787
Participants (n)	152	108
Age (y)	33.5 (6.6)	38.2 (9.9)	0.001
Sex (%)			0.001
Men	78.3	54.6
Women	21.7	45.4
BMI (Kg/m^2^)	27.0 (2.6)	26.4 (3.2)	0.119
Physical activity (%)			0.384
Never	25.0	18.5
1–3 day/week	45.4	52.8
4–7 day/week	29.6	28.7
Energy intake (Kcal/day)	2631 (346)	2430 (351)	0.001
Residence time (y)	25.2 (10.9)	31.9 (12.7)	0.001
Smoking Habit (%)			0.506
Never	93.4	95.4
Smokers	6.6	4.6
Alcohol Consumption (%)			0.088
Never	82.2	89.8
1–5 day/week	17.8	10.2

*Values are presented as mean (SD), except for categorical variables represented in percentage (%). M, meters; n, number of subjects; y, years; Probability p-values from Students' t-test for continuous variables or χ^2^ test of linear trend for categorical variables*.

### Metabolic syndrome and associated components

Living at a higher altitude, both in the crude model and when considering confounding factors, was associated to a lower prevalence of metabolic syndrome and some of its associated components (Table 2). The unadjusted model (crude) displayed lower prevalence of hypercholesterolemia (OR = 0.24; *p* < 0.001), hyperglycemia (OR = 0.24; *p* < 0.01) and MetS (OR = 0.27; *p* < 0.05); and a higher prevalence of hypertension (OR = 5.20; *p* < 0.05). When adjusting for age and sex, a significantly lower prevalence of central obesity (OR = 0.40; *p* < 0.05), hypercholesterolemia (OR = 0.18; *p* < 0.001), hyperglycemia (OR = 0.20; *p* < 0.01) and MetS (OR = 0.16; *p* < 0.01) were found at high altitude. The first multivariate model (Model 1), adjusted for age, sex, self-reported energy intake and residence time showed that participants living at high altitude had a significantly lower prevalence of hypercholesterolemia (OR = 0.22; *p* < 0.001), hyperglycemia (OR = 0.30; *p* < 0.001) and MetS (OR = 0.28; *p* < 0.05). The second multivariate model (Model 2) fitted for age, sex, self-reported energy intake, residence time, BMI, physical activity, smoking habit and alcohol consumption showed that living at high altitude was significantly associated with less hypercholesterolemia (OR = 0.24; *p* < 0.001), hyperglycemia (OR = 0.25; *p* < 0.05) and MetS (OR = 0.24; *p* < 0.05).

**Table 2:** Associations between altitude and prevalence of MetS.

**Elevation**	**Sea level**	**High altitude**
Range (m)	4–6	2,758–2,787
Participants (n)	152	108
**CENTRAL OBESITY**		
Prevalence	135	88
Crude	1.00 Ref.	0.55 (0.28–1.12)
Age and sex	1.00 Ref.	0.40 (0.18–0.87) ^*^
Model 1	1.00 Ref.	1.10 (0.39–3.06)
Model 2	1.00 Ref.	1.32 (0.44–3.98)
**HYPERCHOLESTEROLEMIA**		
Prevalence	69	18
Crude	1.00 Ref.	0.24 (0.13–0.44)^***^
Age and sex	1.00 Ref.	0.18 (0.09–0.36)^***^
Model 1	1.00 Ref.	0.22 (0.11–0.45)^***^
Model 2	1.00 Ref.	0.24 (0.12–0.49)^***^
**HYPERTENSION**		
Prevalence	2	7
Crude	1.00 Ref.	5.20 (1.06–25.53)^*^
Age and sex	1.00 Ref.	2.11 (0.36–12.38)
Model 1	1.00 Ref.	2.56 (0.40–16.43)
Model 2	1.00 Ref.	3.60 (0.49–26.35)
**HYPERGLYCEMIA**		
Prevalence	26	5
Crude	1.00 Ref.	0.24 (0.09–0.63)^**^
Age and sex	1.00 Ref.	0.20 (0.07–0.60)^**^
Model 1	1.00 Ref.	0.30 (0.10–0.92)^***^
Model 2	1.00 Ref.	0.25 (0.07–0.88)^*^
**MetS**		
Prevalence	23	5
Crude	1.00 Ref.	0.27 (0.10–0.74)^*^
Age and sex	1.00 Ref.	0.16 (0.05–0.55)^**^
Model 1	1.00 Ref.	0.28 (0.08–0.95)^*^
Model 2	1.00 Ref.	0.24 (0.07–0.91)^*^

*MetS harmonizing definition (IDF-AHA/NHLBI). Multivariate Model 1 adjusted for: age, sex, self-reported energy intake and residence time. Multivariate Model 2 additionally adjusted for: BMI, physical activity, smoking habit and alcohol consumption; m, meters; n, number of subjects; Ref, category of reference; MetS, metabolic syndrome. Odds Ratios and 95% CI*.

* *p < 0.05*;

** *p < 0.01*;

*** *p < 0.001*.

**Figure 1:**
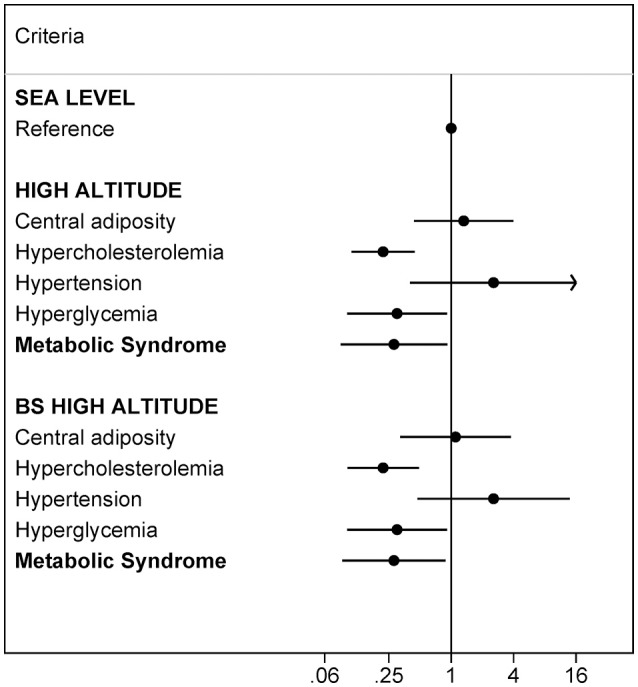
Validity analysis. Risk of developing each component of MetS according to the altitude level assessed with logistic regression and bootstrapped (BS) logistic regression (1,000 random samples). MetS harmonizing definition (IDF-AHA/NHLBI). Multivariate Model 1 adjusted for: age, sex, self-reported energy intake and residence time. Ref. category of reference.

To assess the internal validity of the results, the OR of each condition and the overall MetS were compared to those obtained by the bootstrapping re-sampling method, after adjusting for potential confounding factors as in multivariate Model 1. The analyses showed a similar pattern in the prevalence of MetS and its components, and the variability was reduced in the bootstrapped logistic regression model. At high altitude the bootstrapped analyses showed lower prevalence of hypercholesterolemia (OR = 0.30; *p* < 0.05), hyperglycemia (OR = 0.22; *p* < 0.001) and MetS (OR = 0.28; *p* < 0.05) (Figure 1). In contrast, no association was detected between altitude and the prevalence of central adiposity or hypertension.

**Figure 2:**
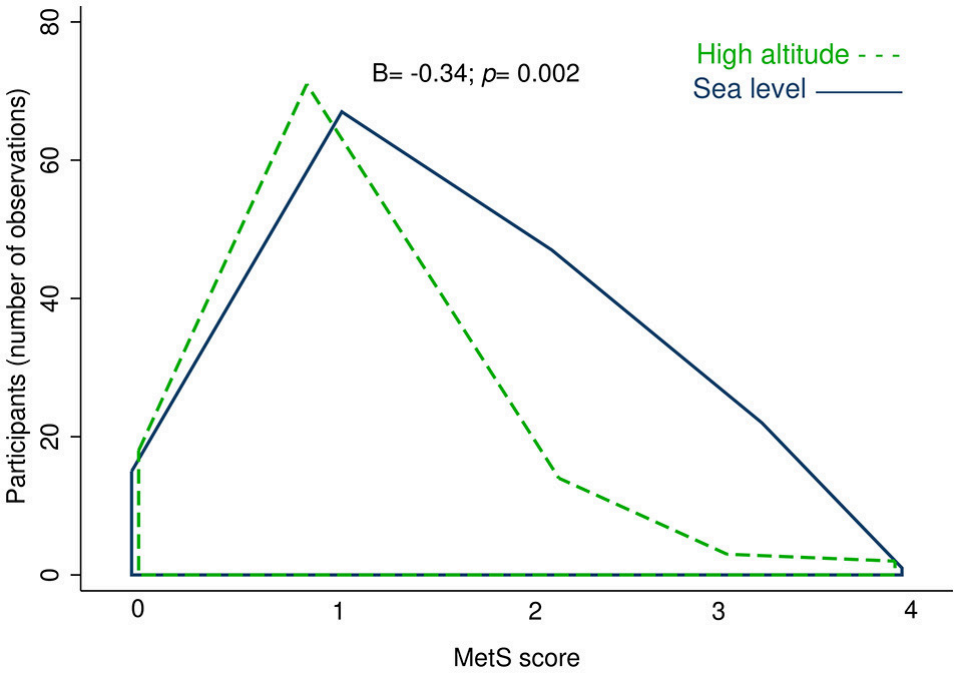
Observed density of people by altitude for the summation of MetS components (MetS score). The high altitude is represented by the area under the green dashed line, and sea level area is displayed under the blue solid line. Model 1 adjusted for: age, sex, self-reported energy intake and residence time. B, Beta regression coefficient; *p*, p-value.

To determine the distribution of MetS components in both altitude levels, a summation of them in a MetS score (0-5 points) was performed. The MetS score showed a reduction in the number of MetS components at high altitude compared to sea level (B = −0.34; *p* = 0.002) (Figure 2).

### Altitude and energy intake

**Figure 3:**
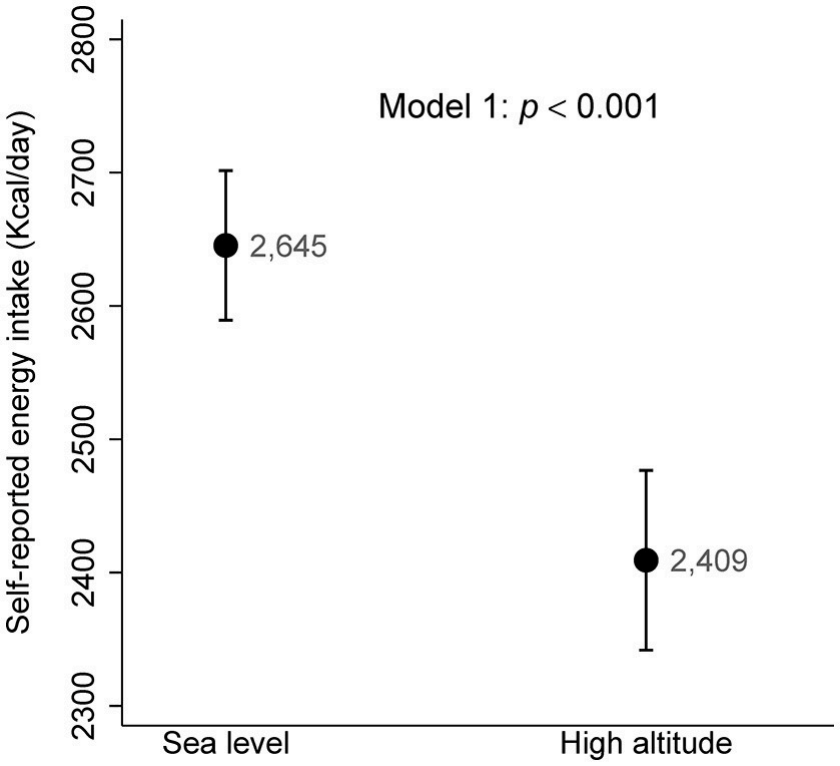
Self-reported energy intake according to the altitude of residence. ANCOVA multivariate Model 1 adjusted for: age, sex and residence time.

The differences observed in self-reported energy intake between the two altitude groups made it not only necessary for the inclusion of this variable in the models, but also pointed to the need of a more exhaustive study, to test whether the appetite was different between those living at high altitude when compared to those living at sea level. A significantly lower self-reported energy intake was found in high altitude compared to sea level, tested through ANCOVA (Figure 3), fitted for age, sex and residence time as Model 1 (*p* < 0.001). To further verify the independent effect of altitude on self-reported energy intake, it was adjusted with the multivariate Model 2 (fitted for: age, sex, self-reported energy intake, residence time, BMI, physical activity, smoking habit and alcohol consumption). This returned a comparable outcome (*p* < 0.001; data not shown), indicating a lower calorie intake at high altitude in both multivariate models.

## Discussion

The present study conducted in a small Ecuadoran population composed of highly educated adults shows that adults living at high altitude (2,758–2,787 m) had lower odds of hypercholesterolemia, hyperglycemia and MetS than those who lived at sea level, adjusting for potential confounders. A similar association was found with the MetS score (summation of each component of MetS), suggesting a reduction in the number of MetS components at high altitude compared to sea level.

To our knowledge, there is only one study on the incidence of MetS at high altitude (12). However, the result was recorded at a moderately high altitude (mean of 635 m), in a Spanish cohort. Moreover, another study relating MetS prevalence at high altitude found no differences between sea level and high altitude, in a population from Peru (18). Despite this, the inverse association between high altitude and the components of MetS was found in previous studies. For example, a study on diabetes and altitude performed on a large dataset, of nearly three hundred thousand adults from the United States, showed lower prevalence of diabetes when living above 1,500 m (10). Categorizing by sex, this study reported that the effect was only statistically significant among men. Moreover, Woolcott et al. (6) also reported that women had lower odds of having obesity and diabetes, as compared with men, regardless of altitude. In our sample, although non-significant interaction (*p* = 0.421) women had 86% lower prevalence of MetS at high altitude compared to men at sea level (OR: 0.14; 95% CI:0.02–0.86), while men at high altitude had 80% lower prevalence to those at sea level (OR: 0.20; 95% CI: 0.04–0.92). Finally, we found that women at sea level had lower prevalence of MetS (OR: 0.26; 95% CI: 0.06–1.23) compared to men at the same altitude (reference) albeit not statistically significant, following a similar trend to that found in Voss et al. (7), where a lower prevalence of obesity was found among women compared to men at lower altitudes. These results suggest that women could be less prone to MetS, as discussed in the aforementioned article (7). Previous studies evidenced lower fasting glycemia (19, 20) and better glucose tolerance (21) at high altitude. Hypercholesterolemia prevalence and hypertension were lower in the high altitude group in Peru (18). The prevalence of central obesity was not associated with altitude in our study. However, another study performed in Andean population reported lower prevalence at high altitude (6). Our results may differ from the aforementioned study in the sample size, as they had a larger cohort, which could increase the power of the study, thus allowing detection of even slight differences. Moreover, other studies carried out in different populations reported lower prevalence (7, 22) and incidence (8, 9) of overweight or obesity at high altitude. On the other hand, a previous study published by our group did not found an association between individual MetS components and altitude (12), suggesting that the association between altitude and chronic diseases is still unclear, and more studies are needed. Some causes of mortality were also associated to altitude, specifically living at high altitude was associated with lower mortality rate from coronary heart disease (23) and ischemia (14), and higher mortality from chronic obstructive pulmonary disease (14). Bootstrapped re-sampling method was used to test the internal validity of logistic regression, as it provides evidence of the consistency of the estimated OR. In general terms, 1,000 or more re-sampled datasets would be enough to calculate CIs with a lower variability (inversely related to the number of re-sampled datasets). In addition to this, the score performed with the summation of MetS components revealed greater degree of comorbidities in those participants living at sea level compared to high altitude subjects.

The inverse association between altitude and self-reported energy intake, while adjusting for covariates, suggested a regulatory role of appetite in MetS reduction even in acclimatized participants living permanently at the same altitude for a median of 29 years. Nevertheless, our results are independent of energy intake as it was used as a potential confounding factor in the multivariate models. Previous studies reported lower appetite at high altitude (hypoxic environment) however most of them have been performed in a short-term time of residence, with subjects from the sea level relocated to high altitude for the purpose of the studies (5). On the other hand, the direct effect of prolonged altitude exposure on appetite remains unknown, therefore, it would be interesting to further investigate the reason. Basal metabolic rate and sympathetic activation does not appear to be higher among highlanders as compared with coast living subjects, even if normalized to fat-free mass (6). Leptin and norepinephrine could be influencing the changes in energy expenditure and food intake at high altitude, by increasing the sympathetic nerve activity and contributing to enhance energy expenditure, remaining even in acclimatized subjects (5, 7, 24). In addition, leptin levels are increased in weight loss at high altitude compared to those at sea level (24).

At sea level the partial pressure of oxygen is approximately 160 mmHg, which corresponds to 21% oxygen respired. The ascent of approximately 300 m of altitude results in a 1% reduction of oxygen concentration (25), but only up to 4,500 m (26). In our study, the subjects lived in their residence for a median time of 29 years (28 years the group at sea level and 30 years those at high altitude). Participants living over 2,700 m have a 27% lower oxygen availability than those at sea level, that means a total partial pressure of oxygen (pO_2_) of 15% in an inhaled gas mixture. The arterial pO_2_ is 13.1 kPa in the group at sea level, and 7.7 kPa at high altitude, which means a 97.3 and 89.6% oxygen saturation in blood, respectively. A reduction in arterial pO_2_ by more than 1 kPa and/or an oxygen saturation below 95% is considered hypoxemia (27); therefore, we could consider participants of the high altitude group (2,758–2,787 m), with a median residence of 30 years, under a chronic hypobaric (low-pressure) hypoxia.

At high altitude there is lower pressure, temperature and humidity (26), therefore other possible explanations apart from reduced oxygen availability cannot be dismissed. In this sense, some studies have found a positive association between temperature and obesity prevalence (28, 29). Moreover, factors such as polymorphisms for high altitude adaptation could be involved in the lower prevalence of MetS and its components at high altitude (16, 17, 30). Some of the genetic variants highly present in high altitude residents are related to hypoxic adaptation (30), while others are related to the antioxidant system and lung function (17).

Our study has several strengths. Firstly, the analyses are adjusted for lifestyle factors to avoid bias. Secondly, all data were collected by a Dietitian-Nutritionist, including waist circumference, allowing central obesity to be diagnosed, reducing data inaccuracy and preventing recall and response biases. Moreover, participants' educational level helped to provide a high quality of self-reported data and adds homogeneity to the cohort regarding socio-demographic factors. Finally, the time of residence (median time of 29 years in our sample) was used for adjustments, which could help avoiding the effect of moving between different altitude levels.

This study should be analyzed in the context of several limitations. Although we have adjusted for potential confounders, we cannot rule out the existence of residual confounding. One should consider reverse causality due to cross-sectional study design. In addition, both the altitude exposure and the outcome are categorized in low/high and presence/absence respectively, but continuous variables would give a more representative outcome. Furthermore, the availability of numerical values could help to identify the exact cut-off point where altitude exerts a favorable association. Bootstrapping method was used to test the internal validity, however it is limited because the sample must be representative of the population from which it was sampled; thus, the smaller the original sample, the less representative to the population (31). This study is focused on population with a university degree; therefore, they might not be representative of the whole population. Finally, Type II error cannot be discarded in cross-sectional studies with targeted subjects. Despite the proximity to the upper limit of the CI to the null value, our results are statistically significant and were in the same line of previous studies.

## Conclusion

The present study concerning a small, Ecuadoran population, composed of highly educated adults living at the coast and the Andean Altiplano with a university degree, revealed that residence at high altitude (2,758–2,787 m) was associated with a lower prevalence of MetS, hypercholesterolemia and hyperglycemia, compared to the participants at sea level (4–6 m). This association was tested with bootstrapping method, which validated our results re-sampling the dataset (1,000 bootstraps). A MetS score calculated with the summation of MetS individual components showed a reduction in the number of MetS components at high altitude compared to sea level, supporting our hypothesis. Finally, an inverse association between altitude and self-reported energy intake was found after adjusting for covariates, suggesting a physiological role of appetite at high altitude even in acclimatized subjects. Nevertheless, future studies with a longitudinal design and larger sample size are needed. The mechanisms involved in the lower prevalence of metabolic disorders should be addressed in a physiological context to clarify the underlying factors of these associations. These findings suggest that altitude may be related not only to MetS but also to some of the individual components.

## Data availability

The datasets generated during the current study are not publicly available due to reasons of confidentiality but are available from the corresponding author on reasonable request.

## Author contributions

PG-M and JAM planned and designed the study, JA data collection, JA and AL-P conducted statistics, JA, AL-P, PG-M, and JAM assisted with interpretation of the results, revised and edited the manuscript, JA and AL-P contributed to writing the original draft. All authors read and approved the final manuscript.

### Conflict of interest statement

The authors declare that the research was conducted in the absence of any commercial or financial relationships that could be construed as a potential conflict of interest.

## References

[1] Grundy SM Cleeman JI Daniels SR Donato KA Eckel RH Franklin BA. Diagnosis and management of the metabolic syndrome: an American heart association/national heart, lung, and blood institute scientific statement. Circulation (2005) 112:2735–52. 10.1161/CIRCULATIONAHA.105.169404 16157765

[2] Kassi E Pervanidou P Kaltsas G Chrousos G. Metabolic syndrome: definitions and controversies. BMC Med. (2011) 9:48. 10.1186/1741-7015-9-48 21542944 PMC3115896

[3] Alberti KG Eckel RH Grundy SM Zimmet PZ Cleeman JI Donato KA. Harmonizing the metabolic syndrome: a joint interim statement of the international diabetes federation task force on epidemiology and prevention; national heart, lung, and blood institute; American Heart Association; World Heart Federation; International. Circulation (2009) 120:1640–5. 10.1161/CIRCULATIONAHA.109.192644 19805654

[4] Anderson JD Honigman B. The effect of altitude-induced hypoxia on heart disease: do acute, intermittent, and chronic exposures provide cardioprotection? High Alt Med Biol. (2011) 12:45–55. 10.1089/ham.2010.1021 21452965

[5] Hirschler V. Cardiometabolic risk factors in native populations living at high altitudes. Int J Clin Prac. (2016) 70:113–8. 10.1111/ijcp.12756 26820389

[6] Woolcott OO Gutierrez C Castillo OA Elashoff RM Stefanovski D Bergman RN. Inverse association between altitude and obesity: a prevalence study among andean and low-altitude adult individuals of Peru. Obesity (2016) 24:929–37. 10.1002/oby.21401 26935008 PMC4814295

[7] Voss JD Masuoka P Webber BJ Scher AI Atkinson RL. Association of elevation, urbanization and ambient temperature with obesity prevalence in the United States. Int J Obes. (2013) 37:1407–12. 10.1038/ijo.2013.5 23357956

[8] Voss JD Allison DB Webber BJ Otto JL Clark LL. Lower obesity rate during residence at high altitude among a military population with frequent migration: a quasi experimental model for investigating spatial causation. PLoS ONE (2014) 9:e93493. 10.1371/journal.pone.0093493 24740173 PMC3989193

[9] Díaz-Gutiérrez J Martínez-González MÁ Pons Izquierdo JJ González-Muniesa P Martínez JA Bes-Rastrollo M. Living at higher altitude and incidence of overweight/obesity: prospective analysis of the SUN cohort. PLoS ONE (2016) 11:e0164483. 10.1371/journal.pone.0164483 27812092 PMC5094724

[10] Woolcott OO Castillo OA Gutierrez C Elashoff RM Stefanovski D Bergman RN. Inverse association between diabetes and altitude: a cross-sectional study in the adult population of the United States. Obesity (2014) 22:2080–90. 10.1002/oby.20800 24890677 PMC4149588

[11] Norboo T Stobdan T Tsering N Angchuk N Tsering P Ahmed I. Prevalence of hypertension at high altitude: cross-sectional survey in Ladakh, Northern India 2007-2011. BMJ Open (2015) 5:e007026. 10.1136/bmjopen-2014-007026 25897026 PMC4410116

[12] Lopez-Pascual A Bes-Rastrollo M Sayón-Orea C Perez-Cornago A Díaz-Gutiérrez J Pons JJ. Living at a geographically higher elevation is associated with lower risk of metabolic syndrome: prospective analysis of the SUN cohort. Front Physiol. (2017) 7:1–9. 10.3389/fphys.2016.00658 28101063 PMC5209344

[13] Burtscher M. Effects of living at higher altitudes on mortality: a narrative review. Aging Dis. (2014) 5:274–80. 10.14336/AD.2014.0500274 25110611 PMC4113517

[14] Ezzati M Horwitz ME Thomas DS Friedman AB Roach R Clark T. Altitude, life expectancy and mortality from ischaemic heart disease, stroke, COPD and cancers: national population-based analysis of US counties. J Epidemiol Commun Health (2012) 66:e17. 10.1136/jech.2010.112938 21406589

[15] Youk AO Buchanich JM Fryzek J Cunningham M Marsh GM. An ecological study of cancer mortality rates in high altitude counties of the United States. High Alt Med Biol. (2012) 13:98–104. 10.1089/ham.2011.1051 22724612

[16] Bigham AW Wilson MJ Julian CG Kiyamu M Vargas E Leon-Velarde F. Andean and Tibetan patterns of adaptation to high altitude. Am J Hum Biol. (2013) 25:190–7. 10.1002/ajhb.22358 23348729

[17] Valverde G Zhou H Lippold S de Filippo C Tang K Lopez Herraez D. A novel candidate region for genetic adaptation to high altitude in Andean populations. PLoS ONE (2015) 10:e0125444. 10.1371/journal.pone.0125444 25961286 PMC4427407

[18] Baracco R Mohanna S Seclén S. A comparison of the prevalence of metabolic syndrome and its components in high and low altitude populations in Peru. Metab Syndr Relat Disord. (2007) 5:55–62. 10.1089/met.2006.0019 18370814

[19] Lindgärde F Ercilla MB Correa LR Ahrén B. Body adiposity, insulin, and leptin in subgroups of Peruvian Amerindians. High Alt Med Biol. (2004) 5:27–31. 10.1089/152702904322963663 15072714

[20] Castillo O Woolcott OO Gonzales E Tello V Tello L Villarreal C. Residents at high altitude show a lower glucose. High Alt Med Biol. (2007) 8:307–11. 10.1089/ham.2007.8407 18081506

[21] Picón-Reátegui E Intravenous glucose tolerance test at sea level and at high altitudes. J Clin Endocrinol Metabol. (1966) 21:1177–80. 10.1210/jcem-23-12-1256 14087605

[22] Sherpa LY Deji Stigum H Chongsuvivatwong V Thelle DS Bjertness E. Obesity in Tibetans aged 30-70 living at different altitudes under the North and South faces of Mt. Everest. Int J Environ Res Publ Health (2010) 7:1670–80. 10.3390/ijerph7041670 20617052 PMC2872340

[23] Faeh D Moser A Panczak R Bopp M Roosli M Spoerri A Group SNCS. Independent at heart: persistent association of altitude with ischaemic heart disease mortality after consideration of climate, topography and built environment. J Epidemiol Commun Health (2016) 70:798–806. 10.1136/jech-2015-206210 26791518

[24] Palmer BF Clegg DJ. Ascent to altitude as a weight loss method: the good and bad of hypoxia inducible factor activation. Obesity (2014) 22:311–17. 10.1002/oby.20499 23625659 PMC4091035

[25] McElroy MK Gerard A Powell FL Prisk GK Sentse N Holverda S. Nocturnal O_2_ enrichment of room air at high altitude increases daytime O_2_ saturation without changing control of ventilation. High Alt Med Biol. (2000) 1:197–206. 10.1089/15270290050144190 11254229

[26] West JB. Commuting to high altitude: value of oxygen enrichment of room air. High Alt Med Biol. (2002) 3:223–35. 10.1089/15270290260131948 12162865

[27] Nielsen HB. Arterial desaturation during exercise in man: implication for O_2_ uptake and work capacity. Scan J Med Sci Sports (2003) 13:339–358. 10.1046/j.1600-0838.2003.00325.x 14617055

[28] Valdes S Maldonado-Araque C Garcia-Torres F Goday A Bosch-Comas A Bordiu E. Ambient temperature and prevalence of obesity in the Spanish population: The Di@bet.es study. Obesity (2014) 22:2328–32. 10.1002/oby.20866 25124468

[29] Yang HK Han K Cho J-H Yoon K-H Cha B-Y Lee S-H. Ambient temperature and prevalence of obesity: a nationwide population-based study in Korea. PLOS ONE (2015) 10:e0141724. 10.1371/journal.pone.0141724 26524686 PMC4629885

[30] Huerta-Sánchez E Degiorgio M Pagani L Tarekegn A Ekong R Antao T. Genetic signatures reveal high-altitude adaptation in a set of ethiopian populations. Mole Biol Evol. (2013) 30:1877–88. 10.1093/molbev/mst089 23666210 PMC3708501

[31] Haukoos JS. Advanced statistics: bootstrapping confidence intervals for statistics with “Difficult” distributions. Acad Emerg Med. (2005) 12:360–65. 10.1197/j.aem.2004.11.018 15805329

